# Drivers of Methotrexate Polyglutamate Concentration in Erythrocytes: Insights from Immune-Mediated Inflammatory Diseases and Pediatric Acute Lymphoblastic Leukemia

**DOI:** 10.3390/ph19020267

**Published:** 2026-02-04

**Authors:** Janani Sundaresan, Wout J. Hamelink, Renske C. F. Hebing, Maartje van de Meeberg, Montse Janssen Bonás, Inge M. van der Sluis, Pascal H. P. de Jong, Martijn Heymans, Robert de Jonge, Maurits C. F. J. de Rotte, Maja Bulatović-Ćalasan

**Affiliations:** 1 Laboratory of Specialized Diagnostics & Research, Department of Laboratory Medicine, Amsterdam University Medical Center, 1105 AZ Amsterdam, The Netherlands; j.sundaresan@amsterdamumc.nl (J.S.); m.derotte@amsterdamumc.nl (M.C.F.J.d.R.); 2 Department of Pharmacy, Sint Maartenskliniek, 6574 NA Nijmegen, The Netherlands; 3 Department of Gastroenterology and Hepatology, Amsterdam University Medical Center, Amsterdam Gastroenterology Endocrinology Metabolism Research Institute, 1105 AZ Amsterdam, The Netherlands; 4 Department of Gastroenterology and Hepatology, University Medical Center Utrecht, 3584 CX Utrecht, The Netherlands; 5 ILD Center of Excellence (Member of the European Reference Network-Lung), St. Antonius Hospital, 3435 CM Nieuwegein, The Netherlands; 6 Princess Máxima Center for Pediatric Oncology, 3584 CS Utrecht, The Netherlands; 7 Department of Rheumatology, Erasmus MC, 3015 GD Rotterdam, The Netherlands; 8 Department of Epidemiology and Data Science, Amsterdam UMC, Vrije Universiteit Amsterdam, 1081 HV Amsterdam, The Netherlands; 9 Department of Rheumatology & Clinical Immunology, University Medical Center Utrecht, 3584 CX Utrecht, The Netherlands

**Keywords:** pharmacokinetics and drug metabolism, methotrexate polyglutamate, immune-mediated inflammatory diseases, pediatric acute lymphoblastic leukemia, multivariate linear regression modelling

## Abstract

**Background/Objectives**: Methotrexate (MTX) is a cornerstone drug used to treat immune-mediated inflammatory diseases (IMIDs) in low doses (10–30 mg/week), and malignancies in high doses (5000 mg/m^2^/2 weeks). Its active metabolites, Methotrexate polyglutamates (MTX-PG_2–5_), quantified in erythrocytes, are associated with efficacy. This study aimed to compare erythrocyte MTX-PG concentrations in patients with IMIDs and pediatric acute lymphoblastic leukemia (ped-ALL) treated with low-dose or high-dose MTX, respectively, and to identify clinical, demographic, and treatment-related factors influencing their concentration. **Methods**: A total of 567 patients with rheumatoid arthritis, juvenile idiopathic arthritis, Crohn’s disease, sarcoidosis, and ped-ALL were included. Erythrocyte MTX-PG concentration data was collected after 3 months (2.5 months for ped-ALL patients) of MTX-use. Multivariate linear regressing modelling adjusting for age, sex, body mass index (BMI), smoking status, starting MTX dose, route of MTX administration, use of predniso(lo)ne, disease-modifying anti-rheumatic drugs (DMARDs), and folic (or folinic) acid was performed. **Results**: Intravenous high-dose MTX increased MTX-PG_4&5_ accumulation. Despite 50-fold higher doses in ped-ALL, MTX-PG_2–5sum_ concentrations were similar to those seen with subcutaneous low-dose MTX used in IMIDs. Age positively influenced MTX-PG concentrations, while DMARD use reduced MTX-PG_2–3&5_ concentrations. Interestingly, predniso(lo)ne use was associated with higher MTX-PG_4&5_ concentrations and folic (or folinic) acid with higher MTX-PG_3–5_ concentrations. **Conclusions**: This is the first study to compare erythrocyte MTX-PG concentration in low-dose and high-dose patients. Intravenous high-dose MTX administration increased long-chain MTX-PG_4&5_ concentrations, with MTX-PG_2–5sum_ concentrations similar compared to low-dose subcutaneous MTX use. This study demonstrated that route of administration, age, and concomitant therapies such as DMARDs, predniso(lo)ne, and folic (or folinic) acid significantly influence MTX-PG concentrations.

## 1. Introduction

Methotrexate (MTX) is an antifolate with dose-dependent pharmacologic effects: low dose (maximum of 30 mg/week) acts an anti-inflammatory drug, while low and high doses (2–12 g/m^2^/dose) are employed in chemotherapy [1,2]. Efficacy, good safety profile, and low costs make low-dose MTX a cornerstone treatment in immune-mediated inflammatory diseases (IMIDs). MTX is the first-line treatment in rheumatoid arthritis (RA), juvenile idiopathic arthritis (JIA), and next-in-line treatment in Crohn’s disease (CD), sarcoidosis, and other IMIDs like psoriasis and atopic dermatitis [3,4,5].

The native form of MTX contains a glutamate moiety. Intracellularly, MTX is metabolized to its active forms—Methotrexate polyglutamate (MTX-PG)—by folyl-polyglutamate synthetase (FPGS), which adds up to five glutamate moieties (MTX-PG_2–5_). This enhances cellular retention [6], and can inhibit key enzymes involved in folate metabolism, resulting in either anti-inflammatory effects, via adenosine production in IMIDs [2,6], or chemotherapeutic effects, via blockade of de novo purine and thymidine synthesis pathways in malignancies [6].

As early as one month after starting low-dose MTX, MTX-PGs have been quantified in erythrocytes [7]. Quantifying these metabolites can aid in therapeutic drug monitoring (TDM) as erythrocytes are easily obtained with a blood withdrawal. Secondly, erythrocyte MTX-PGs have a well-established efficacy relationship. A recent meta-analysis of 21 studies including inflammatory arthritis, dermatitis, and colitis demonstrated a consistent positive association between concentrations of the sum of individual MTX-PGs and the efficacy of MTX treatment [3]. A recent MTX-PG exposure–response model in 395 RA patients suggested that after 3 months of MTX use, the cumulative concentration of the long-chain MTX-PGs (MTX-PG_3–5sum_) between 47 and 78 nmol/L of packed erythrocytes represented an optimal therapeutic range [8]. Lastly, erythrocyte MTX-PGs demonstrated inter- and intra-individual variations. In contrast to IMIDs, studies correlating erythrocyte MTX-PG concentrations and efficacy in pediatric acute lymphoblastic leukemia (ped-ALL) are lacking.

Prior investigations examining factors affecting MTX-PG levels identified demographic (age, sex), lifestyle (smoking and alcohol consumption), genetic (SNPs in genes encoding enzymes of the folate pathway), and clinical (kidney and liver function) factors [9,10,11]. These studies, however, were performed primarily in RA patients, receiving low-dose MTX. In this work, we aim to expand the current knowledge by investigating clinical, demographic, and treatment-related factors that influence erythrocyte MTX-PG concentration across diseases, by including IMID patients on low-dose (RA, JIA, CD and sarcoidosis) and ped-ALL patients on high-dose MTX therapy. This is the first study to identify common factors that influence MTX-PG concentration across distinct disease settings, including a cohort of patients on high-dose therapy. Inclusion of the high-dose ped-ALL cohort allowed us to assess whether these determinants remain consistent or show saturation at higher exposures, which clarifies the dose robustness of potential TDM markers. Understanding these determinants may strengthen TDM by improving the prediction of MTX efficacy and toxicity from MTX-PGs, thus, supporting more personalized dosing strategies.

## 2. Results

### 2.1. Basic Patient Characteristics

Baseline characteristics of the 567 patients included in the analysis, stratified by disease, are summarized in Table 1. The mean (SD) age of the patients was 41.59 (22.63) and was significantly different (*p* < 0.0001) between the pediatric (JIA and ped-ALL) and adult cohorts (RA, CD, sarcoidosis). The proportion of female patients exceeded 50% in the RA, JIA, and CD cohorts, whereas sarcoidosis and ped-ALL included fewer than 41% females. The starting doses of MTX in IMIDs were significantly lower than in ped-ALL (*p* < 0.0001). Amongst the IMID cohorts, CD patients were administered the highest starting MTX dose of 25 mg/week, and it was all administered subcutaneously. Oral MTX was given in 91.3% of RA and 98.3% of JIA patients. At baseline, combination DMARD therapy was used in 47% of RA patients as opposed to other IMIDs (*p* < 0.001).

### 2.2. MTX-PG Concentrations Stratified by Diseases

**Figure 1:**
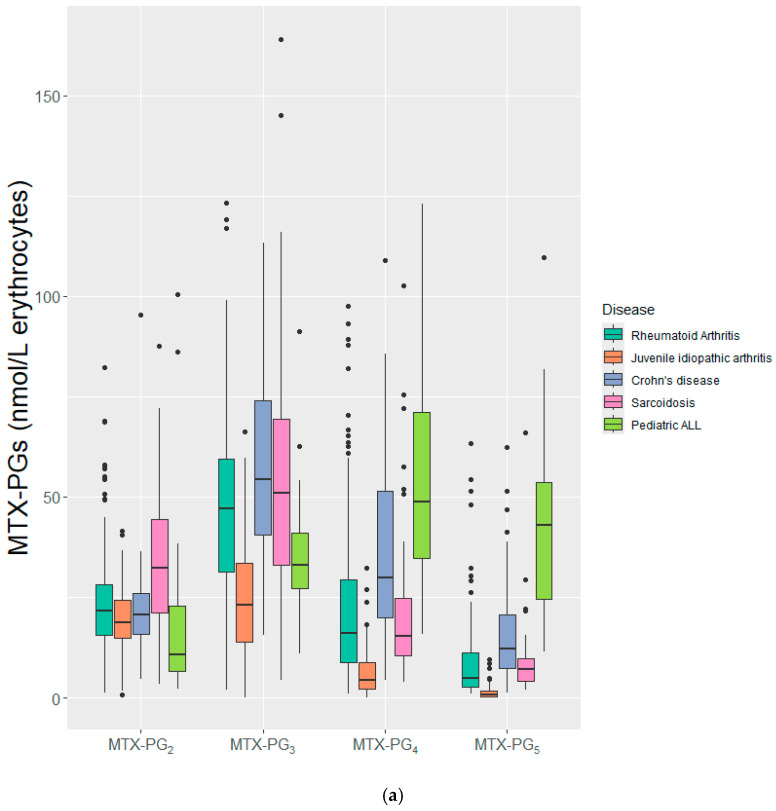

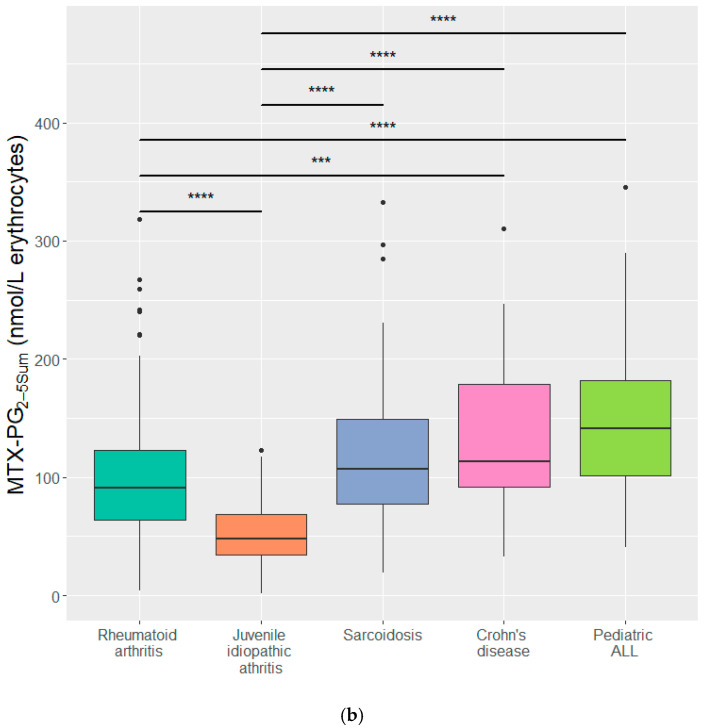
Concentration of (**a**) individual MTX-PGs and (**b**) MTX-PG_2–5sum_ after 3 months of MTX treatment (2.5 months for ped-ALL) stratified by disease. IMIDs were treated with low-dose (5–30 mg/week) oral/S.C., MTX while ped-ALL patients were treated with high-dose (5000 mg/m^2^/2 weeks) I.V./I.Th MTX. IMIDs: Immune-mediated inflammatory diseases, MTX: Methotrexate, S.C.: subcutaneous, I.V./I.Th.: Intravenous/intrathecal, Ped-ALL: Pediatric acute lymphoblastic leukemia, MTX-PG: Methotrexate polyglutamate. *** *p*-value ≤ 0.001, **** *p*-value ≤ 0.0001.

Individual MTX-PGs and MTX-PG_2–5sum_ were quantified after 3 months of MTX use in IMIDs and after approximately 2.5 months after start of treatment in ped-ALL. The overall median (IQR) concentrations of individual MTX-PG_2–5_ were 21.62 (15.51–28.14), 41.3 (26.32–57.4), 15.42 (6.81–29.97), and 5.3 (1.9–12.04) nmol/L, respectively. The overall median (IQR) concentration of MTX-PG_2–5sum_ was 88.71 (55.16–124.86) nmol/L and 63.3 (34.76–97.49) nmol/L for MTX-PG_3–5sum_ (Table S1). Remarkably, despite an approximately 50-fold higher dose, the MTX-PG_2–5sum_ concentration in the ped-ALL cohort was not significantly different from that in CD and sarcoidosis (Figure 1 and Figure S1; Table S1). The JIA cohort had the lowest concentrations of MTX-PG_2–5_ among all the study cohorts.

### 2.3. MTX-PG Concentrations Stratified by Route of Administration and Starting MTX Dose

**Figure 2:**
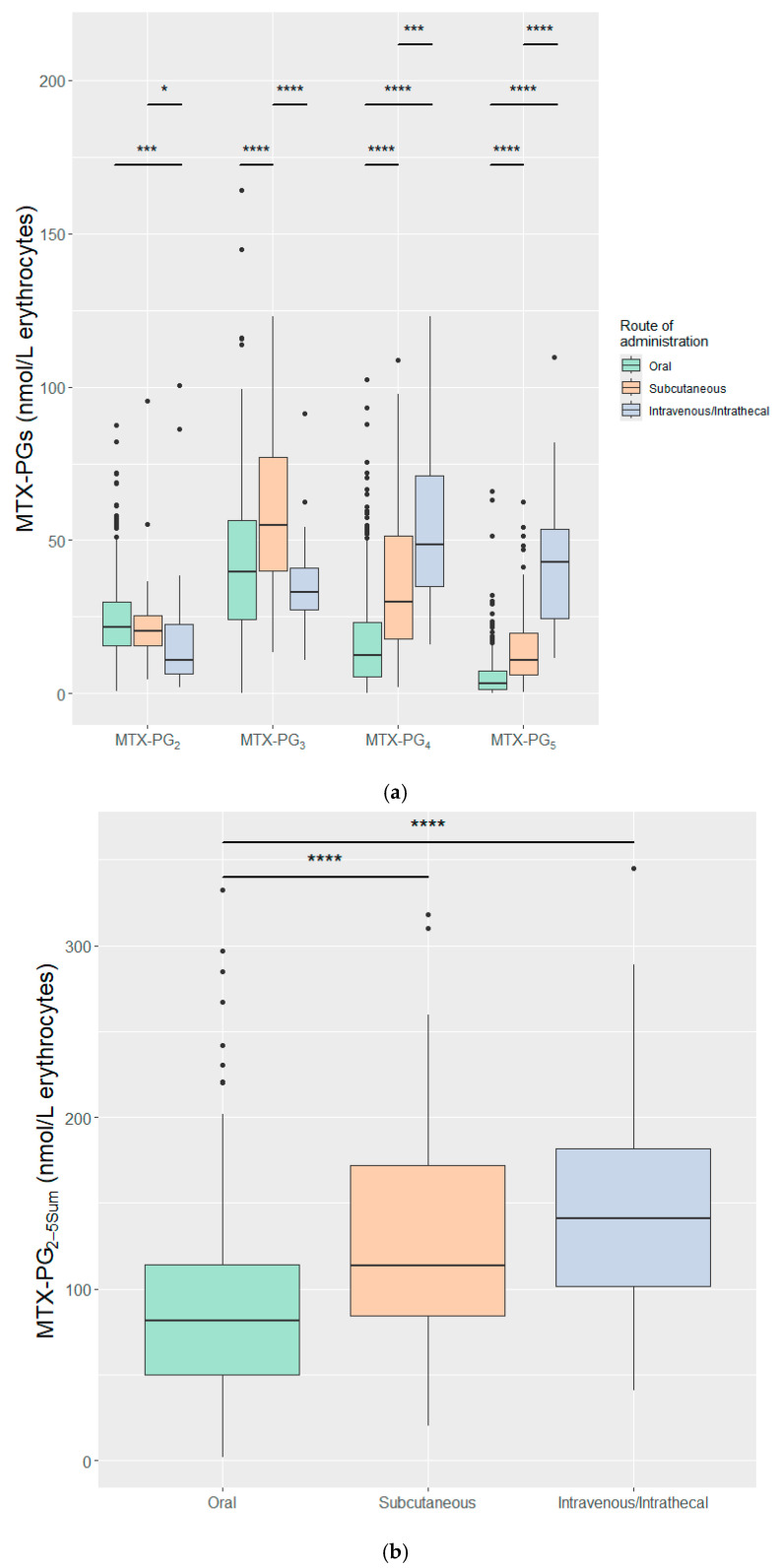

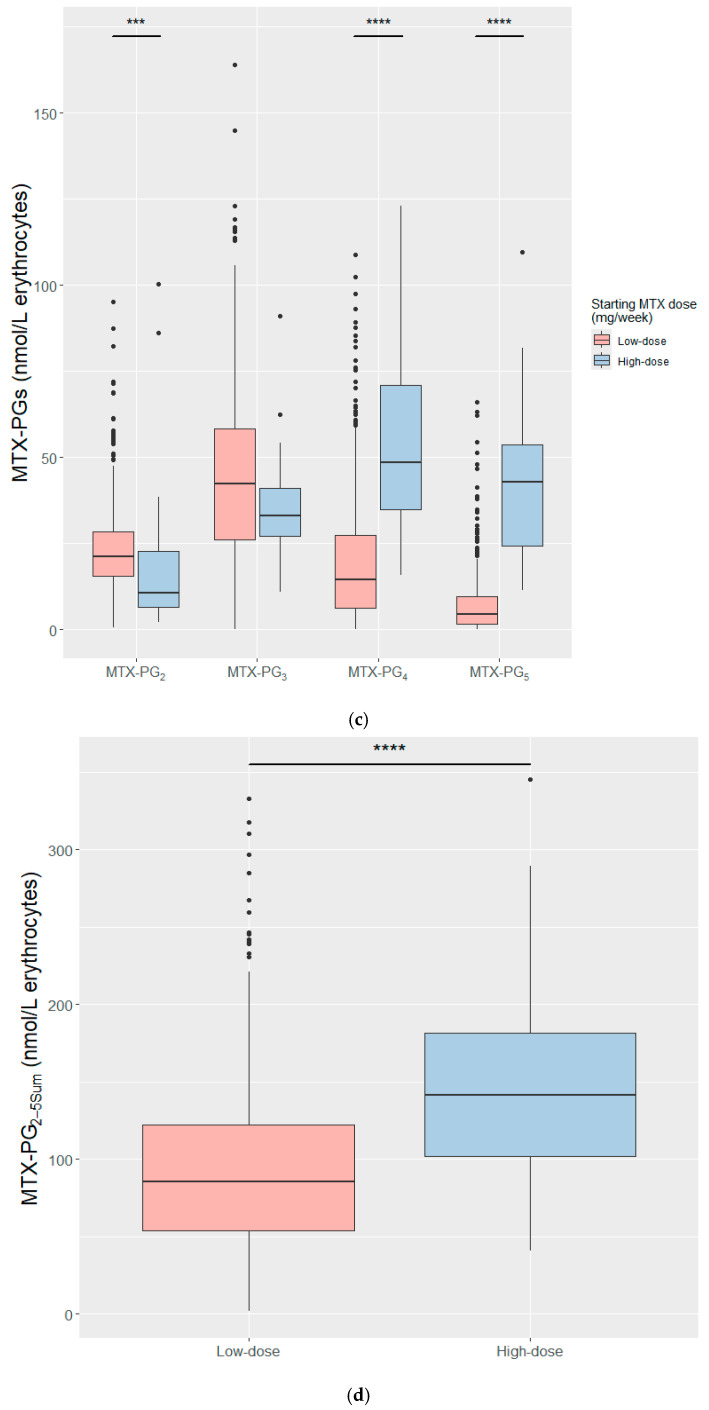
Concentration of individual MTX-PGs and MTX-PG_2–5sum_ after 3 months of MTX treatment in IMIDs or 2.5 months in ped-ALL. (**a**,**b**) Categorized based on route of MTX administration and (**c**,**d**) by dose. Oral and subcutaneous routes were administered low-dose (10–30 mg/week) and intravenous/intrathecal was administered high-dose MTX (5000 mg/m^2^/2 weeks). Ped-ALL: Pediatric acute lymphoblastic leukemia, MTX-PG: Methotrexate polyglutamate. * *p*-value < 0.05, *** *p*-value ≤ 0.001, **** *p*-value ≤ 0.0001.

Route of administration has a direct impact on MTX bioavailability and, thus, the concentration of MTX-PGs. Stratified based on the route of administration, Figure 2a,b depicts individual MTX-PG and MTX-PG_2–5sum_ concentrations per three categories of oral, S.C., and I.V./I.Th. administrations. I.V./I.Th. administration, given exclusively to ped-ALL patients receiving high-dose MTX, resulted in significantly higher concentrations of MTX-PG_4–5_ compared with S.C. and oral routes in low-dose IMID patients, whereas S.C. administration showed significantly higher concentration of MTX-PG_3_ than I.V./I.Th. and oral routes (Figure 2a). Interestingly, MTX-PG_2–3_ concentrations were significantly lower following I.V./I.Th. administration compared to oral and S.C. routes. Despite differences in individual MTX-PGs concentrations, MTX-PG_2–5sum_ was not significantly different between high-dose I.V/I.Th and low-dose S.C. MTX administrations but was significantly higher than in low-dose MTX oral administration (Figure 2b). When stratifying based on dose, high-dose MTX favoured MTX-PG_4&5_ concentrations (Figure 2c) and the MTX-PG_2–5sum_ after high-dose MTX was significantly higher than after low-dose MTX treatment (Figure 2d). Median MTX-PG_2–5sum_ was similar between the I.V./I.Th. [141.3 (40.5–345.2)] and S.C. [113.64 (20.37–317.9)] categories.

### 2.4. Model Diagnostics

To assess the model assumption and the adequacy of the imputation process, we performed a series of diagnostic evaluations. Because disease exhibited substantial multicollinearity with route of administration (variance inflation factor > 5), it was excluded from the final regression models and consequently, analyses requiring grouping or stratification by disease were not performed. Prior to imputation, missingness patterns were assessed. Four columns had missing values (<30%; Figure S2). A list of variables and auxiliary variables is given in Table S2. Stripplots comparing imputed and observed values for the imputed variables indicated good agreement (Figure S3). Exploratory residual analysis did not reveal major deviations from model assumptions and representative residual plots are shown in Figure S4.

### 2.5. Determinants of MTX-PG Concentrations

**Figure 3:**
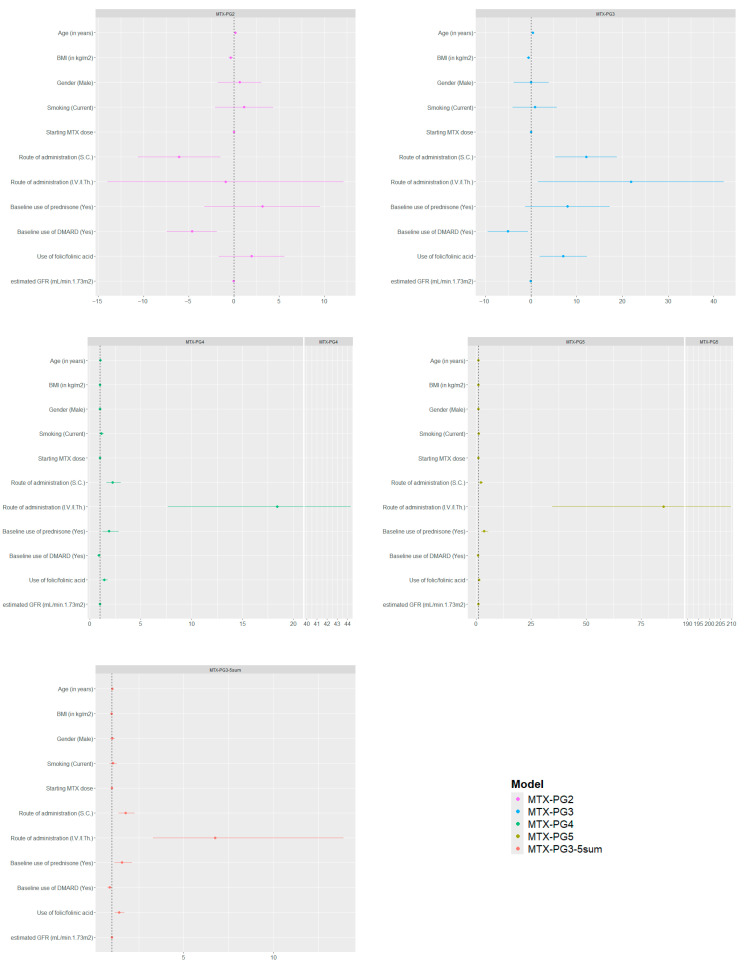
The regression coefficients of the idividual MTX-PGs were plotted as a forest plot. MTX-PG: Methotrexate polyglutamates, BMI: Body mass index, S.C.: Subcutaneous, I.V./I.Th.: Intravenous/intrathecal, DMARD: Disease-modifying anti-rheumatic drug, GFR: Glomerular filtration rate.

In our multivariate linear regression models, age was strongly associated with individual MTX-PG concentrations. Higher age associated with increased MTX-PG_4–5_ [β (*p*-value): 1.01 (0.003), 1.01 (0.002) respectively] concentrations, with a weaker but positive association for MTX-PG_2–3_ [β (*p*-value): 0.16 (0.01), 0.36 (*p* < 0.001), respectively]. In contrast, BMI demonstrated negative associations with MTX-PG_2–3_ concentrations, most prominently with MTX-PG_3_ [β (*p*-value): −0.61 (0.002)]. While MTX-PG_4–5_ were not significantly associated, MTX-PG_4_ showed a non-significant trend [e^β^ (*p*-value): 0.98 (0.06)] (Table 2, Figure 3).

Within the same multivariate models, concomitant therapies were associated with variability in MTX-PG concentrations. Folic/folinic acid supplementation (>5 mg/week) was positively associated with MTX-PG_3–5_ concentrations, particularly with MTX-PG_3_ [β (*p*-value): 7.07 (0.008)], while MTX-PG_2_ showed no association. Baseline predniso(lo)ne use was positively associated, MTX-PG_4–5_ [e^β^ (*p*-value): 1.83 (0.007) and 3.65 (*p* < 0.001), respectively], suggesting corticosteroids may facilitate increase in concentrations of longer-chain MTX-PG_4–5_. In contrast, baseline DMARD (hydroxychloroquine (HCQ) ± sulfasalazine (SSZ)) use was negatively associated with MTX-PG_2–3_ [β (*p*-value): −4.62 (0.001) and −5.25 (0.02), respectively], and modestly with MTX-PG_5_ [e^β^ (*p*-value): 0.78 (0.02)], while MTX-PG_4_ showed no association, suggesting that baseline DMARD use may selectively influence MTX-PG concentrations, which could in turn affect treatment efficacy or toxicity profiles (Table 2, Figure 3).

Within the same multivariate models, we examined the effects of the routes of administration and starting MTX dose after adjusting for other covariates. The S.C and I.V./I.Th. routes of administration demonstrated positive associations with longer-chain MTX-PGs. S.C. route was associated with higher MTX-PG_3–5_ concentrations [β (*p*-value): 11.84 (0.001); e^β^ (*p*-value): 2.25 (*p* < 0.001) and 2.19 (*p* < 0.001), respectively], while the short-chain MTX-PG_2_ [β (*p*-value): −5.77, (*p* = 0.008)] demonstrated lower concentrations. Similarly, higher MTX-PG_3–5_ levels were strongly associated with the I.V./I.Th. route [β (*p*-value): 22.26 (0.04); e^β^ (*p*-value): 18.65 (*p* < 0.001) and 86.48 (*p* < 0.001), respectively], while MTX-PG_2_ was not significantly associated (Table 2, Figure 3). The starting MTX dose was not significantly associated with MTX-PG_2–5_ concentrations (Figure 3). Interestingly, in the subset of IMID patients, higher starting dose was associated with modestly lower MTX-PG_2_ concentration [β (*p*-value): −0.53 (*p* < 0.001)] (Table S3). Extending the analysis by replacing the cumulative MTX dose instead of starting dose in the multivariate model demonstrated no significant associations with MTX-PG_2–5_ concentrations (Table S4).

The other covariates within our models, such as sex, smoking status, and eGFR were not associated with individual MTX-PG concentrations (Table 2, Figure 3, Figure S3 and Figure S4). Model performance varied across metabolites. The model for MTX-PG_5_ explained the greatest variance (R^2^: 0.58), while MTX-PG_2_ was the least well explained (R^2^: 0.09) (Table 2).

In our multivariate linear regression model for MTX-PG_3–5sum_, the route of administration remained the strongest positively associated factor within the model for I.V./I.Th. [e^β^ (*p*-value): 6.75 (*p* < 0.001)] and S.C. [e^β^ (*p*-value): 1.75 (*p* < 0.001)]. Similar to individual MTX-PG models, baseline predniso(lo)ne use and higher folic/folinic acid (>5 mg/week) were positively associated [e^β^ (*p*-value): 1.59 (0.004) and 1.40 (*p* < 0.001), respectively] and BMI negatively associated with MTX-PG_3–5sum_ concentration [e^β^ (*p*-value): 0.98 (*p* = 0.001)], while use of DMARDs showed a non-significant negative trend [e^β^ (*p*-value): 0.87 (0.07)]. The model explained modest variance with an R^2^ of 0.32 (Table 2).

## 3. Discussion

Studies examining determinants of erythrocyte MTX-PG concentrations have predominantly been disease-specific, focusing mainly on RA [9,10,11,12,13]. To our knowledge, this is the first study that combined IMID patients (on low-dose oral/S.C. MTX) and ped-ALL patients (on high-dose I.V./I.Th. MTX) to identify factors that associated with erythrocyte MTX-PG concentrations. We identified the factors that determined the concentrations of individual erythrocyte MTX-PGs and MTX-PG_3–5sum_ concentrations after 3 months of MTX use.

We observed the characteristic pattern of individual MTX-PG concentrations in both individual diseases and in the combined IMID cohorts [10,12]. Among all cohorts, we noticed the lowest levels of MTX-PGs in the JIA cohort, possibly due to a combination of factors like lower MTX dose and younger age of the patients compared to the other cohorts. Oosterom et al. demonstrated detectable concentrations of erythrocyte MTX-PGs before the first I.V. course in ped-ALL patients treated with intrathecal MTX in chemotherapy, reflecting the crossing of MTX from the intrathecal compartment to the systemic circulation [14]. Because of this systemic exposure, the concentration of MTX-PGs arising from I.Th. administration cannot be distinguished from that resulting from the subsequent I.V. dose. Consequently, I.V. and I.Th. routes were analyzed together. The concentration pattern in high-dose ped-ALL patients shifted towards increased MTX-PG_4–5_ levels (Figure S1). Strikingly, despite the prescription of approximately 50-fold higher I.V./I.Th. dose in ped-ALL, MTX-PG_2–5sum_ concentration was similar to that observed in subcutaneous low-dose MTX in IMIDs, while favouring increased longer-chain MTX-PG_4–5_ concentrations. A previous study in RA showed that increasing only the MTX dose (without change in the administration route) resulted in an increased concentration of MTX-PG_4–5_ [10]. Among the different routes, I.V./I.Th. and S.C. have higher bioavailability compared to the oral route [15]. Studies have demonstrated that I.Th. MTX can enter systemic circulation [16]. However, the bioavailability of I.Th. MTX has not been characterized. Due to the study design, the proportion of MTX-PG accumulation due to I.Th. MTX could not be distinguished from the proportion of MTX-PG accumulated due to the I.V. MTX. and thus, cannot be taken fully into account. Thus, the I.Th. route was considered together with the I.V. route. Similar MTX-PG_2–5sum_ concentrations observed between S.C. and I.V./I.Th. administration might be due to either saturation of the uptake receptor RFC (reduced folate carrier) on the erythrocyte precursors cells or the FPGS activity acting as rate-limiters [14,17]. As anticipated, oral route resulted in the lowest levels of MTX-PGs possibly attributable to a lower bioavailability amongst the routes [15].

A population pharmacokinetics–pharmacodynamics study modelling MTX-PGs and therapy efficacy validated the importance of MTX-PG_3–5sum_ in RA patients, while also identifying a therapeutic cut-off for MTX-PG_3–5sum_ concentrations [8]. We modelled the factors that affected the concentration of MTX-PG_3–5sum_ due to its significance in relation to therapy efficacy.

Across different cohorts, age had a consistent positive effect on the accumulation of MTX-PGs and MTX-PG_3–5sum_, in line with the literature on RA patients [3,9,10,18]. This suggests increased concentrations of MTX-PGs in older patients compared to younger patients. Morgaceva et al. suggested that the lower intracellular water volume and reduced phase-I metabolism could lead to increased MTX-PG concentrations and increased half-life of MTX in older patients [19]. Similarly, BMI showed a consistent negative association with concentrations of MTX-PG_2–3_ and MTX-PG_3–5sum_ in our models. Hydrophilic drugs have a smaller volume of distribution that correlated with lean mass [20], and obesity in women was associated with elevated inflammatory markers [21]. Higher BMI was a risk factor for poor response to MTX, increasing the need for higher MTX dosing, especially in established RA patients [22,23,24]. Cross-sectional studies in CD and RA patients suggested no association between BMI and MTX-PGs concentrations [9,18]. However, this contrasted our models, which identified BMI as having a negative association with MTX-PG_3–5sum_ concentration. Given the association between MTX-PG_3–5sum_ and MTX therapy efficacy, our models suggest the need to re-evaluate dosing in patients with higher BMI. It is possible that despite receiving the standard MTX dose, these patients are still sub-optimally dosed, affecting their response to treatment and quality of life. These patients would thus require higher MTX dose or a change to S.C. route, to achieve therapeutic efficacy.

We did not find associations between sex, smoking, and MTX-PG concentrations. Literature on the association between sex and MTX-PG concentrations is contradictory with some studies showing a positive association between females and MTX-PG_2–5_ concentrations [23], while others suggest no association [9,11]. One study in pediatric and adolescent ALL patients on maintenance dose of MTX (low-dose) showed that females tend to have lower erythrocyte MTX-PG_3_ compared to males [25]. Smoking data was not collected in our paediatric cohorts and consequently they were considered non-smokers due to their age, which could have contributed to the non-association of smoking. Previous studies, mainly in RA have associated smoking or the number of cigarettes with reduced MTX response [26,27,28]. Our group developed a Methotrexate non-response prediction model for RA patients, which established smoking as a key predictor of non-response [28]. A retrospective study with 92 CD patients on MTX maintenance treatment, reported that current smokers had a higher risk of relapse (HR = 1.91, 95% CI: 1.11–3.27) [29]. Our current models do not associate smoking with MTX-PG concentrations. This suggest that smoking does not impact MTX-PGs but could exert its negative influence on efficacy through other factors. Kidney function, estimated via eGFR, was the only clinical factor included in our models and was not significant. The literature on eGFR is inconsistent, with studies reporting either a negative association [9,18] or no association [11,30]. As age influences eGFR, the wide age range of patients may have masked any potential effect of kidney function.

Route of administration and dose of MTX determine plasma bioavailability of MTX, influencing MTX-PG concentrations. Oral MTX has a lower bioavailability compared to parenteral administration routes [15,31]. Our models demonstrated significantly elevated MTX-PG_4–5_ and MTX-PG_3–5sum_ accumulation by I.V./I.Th., followed by S.C. administration after correcting for dose and other covariates. This must be considered with caution as the route of administration could have partially masked dose-related effects on MTX-PG concentration. Prior studies suggested no advantage of S.C. compared to oral administration after 3 months of MTX use [7,32]. However, an initial increase in MTX-PG_3–5sum_ concentration by 3 months could be achieved with S.C. administrations. Starting MTX dose negatively affected the MTX-PG_2_ concentration in the subset of IMID patients possibly because of the push towards longer-chain MTX-PG_4–5_ at higher doses. Although the cumulative MTX dose differed between the cohorts due to different escalation schemes, additional analysis using cumulative dose over 3 months (or 2.5 months in ped-ALL) also did not significantly associate with individual MTX-PG concentration consistent with the primary models, confirming the MTX-PG concentrations were not significantly influenced by differences in cumulative exposure. Prior studies have shown that the erythrocyte MTX-PG concentrations reach steady state after 3 months of MTX use [7,32]. The timing of sample collection could influence MTX-PG_1_ concentrations (native form of MTX) but not MTX-PG_2–5_ (unpublished data). In our IMID cohorts, blood was sampled within a week after the previous MTX dose, while in ped-ALL this was two weeks after the last I.V. dose. This timing was not expected to have an impact on the MTX-PG_2–5_ concentrations, as we have previously shown that MTX-PGs do not decline substantially within the first month after stopping MTX treatment [33]. Due to this, we did not expect the MTX-PG_2–5_ concentrations to be affected by sampling time.

The effects of concomitant therapies (DMARDs or predniso(lo)ne) were analyzed separately. DMARD use was considered an RA-specific category, comprising HCQ with or without SSZ and the combined effects on MTX-PG_2–5_ concentrations were analyzed. Previous studies reported a significant negative association between DMARD use and MTX-PG_2_ in RA patients on long-term MTX [9]. In our analysis, we confirmed these negative associations for MTX-PG_2–3&5_ in the full dataset and MTX-PG_4–5_ and MTX-PG_3–5sum_ in the IMID cohorts. Despite different targets, SSZ and MTX share the reduced folate carrier uptake transporter and binding of SSZ to RFC inhibits MTX uptake [34]. We could not investigate the effects of SSZ with MTX combination as most of our RA patients were on the triple therapy of MTX with HCQ and SSZ. Despite the wide use of predniso(lo)ne-MTX combination, the mechanism of influence is hitherto not clearly understood. A previous study found that predniso(lo)ne use resulted in higher MTX-PG_2–4_ and MTX-PG_3–5sum_ levels in RA patients [9]. Our models in both the full cohort and in the IMID patient subset indicate increased MTX-PG_4&5_ and MTX-PG_3–5sum_ concentrations in predniso(lo)ne users. A plausible mechanism is via inhibition of aldehyde oxidase (AO), a liver enzyme responsible for oxidizing MTX to 7-hydroxy-MTX. This interaction can hypothetically increase the availability of MTX in plasma leading to an increase in MTX-PG levels [35].

Folic/folinic acid supplementation can mitigate the toxicity of MTX treatment; however, there is no consensus on the dose. As MTX and folic acid share transporters, folic/folinic acid is usually prescribed after MTX intake/infusion (after 24 h in IMIDs or 42 h in ped-ALL). Our model suggests the use of folic/folinic acid increases concentrations of MTX-PG_3–5_ and MTX-PG_3–5sum_ in the full cohort and MTX-PG_2–4_ and MTX-PG _3–5sum_ in the IMIDs cohort. Similar to prednisone, folic acid is also an inhibitor of AO leading to decreased MTX clearance [35,36]. The decreased clearance might contribute to increased concentrations of MTX-PG_1–5_. Additionally, this could also be reflective of the FPGS activity of erythrocytes during their immature stages. Increasing folic acid doses from 0.5 to 30 mg/week did not have increased advantages and can increase plasma unmetabolized folic acid, leading to other medical concerns [37,38,39]. Associations between predniso(lo)ne use and dosage of folate supplementation should be interpreted with care, as, in routine clinical care, these factors may reflect disease characteristics, treatment variables, or organ function that can also influence MTX PG levels.

One of the main strengths of this study is that MTX-PGs were quantified with the same method using a UPLC-MS/MS, limiting methodological bias. Secondly, incorporating multiple diseases helped identify factors that were disease independent. The study has some limitations as well. Firstly, the cohorts had some missing data, which was handled using the robust multiple imputation method. Secondly, the effect of DMARD comedication on MTX-PG concentrations was predominantly an “RA-category” and the effects of HCQ and SSZ were not investigated separately as most patients were on triple-therapy with MTX, HCQ, and SSZ. Furthermore, use of predniso(lo)ne and folic acid was protocolized and was, therefore, independent of inflammation status; thus, inflammation status was not included in the models. I.V. and I.Th. administrations were analyzed together due to the crossing of intrathecal MTX into systemic circulation, exposing erythrocytes to MTX prior to the I.V. doses. As disease was collinear with the route of administration in our dataset, we were unable to include interaction terms or perform stratified analysis without compromising model stability. Finally, since in CD and sarcoidosis MTX is a next-in-line therapy, the effects of number and types of prior therapies were not considered as cofactors [33].

The R^2^ of all our models was between 0.09 and 0.58, with MTX-PG_5_ having the highest R^2^ of 0.58. In line with prior literature, a large amount of variation not determined by the factors was included in our models [9,10,11]. Notably, in line with the literature, the lowest R^2^ was observed for the shorter-chain MTX-PG_2–3,_ as these are highly influenced by other factors, including uptake/efflux transporter expression and function, bioavailability of plasma MTX or gut microbiome [40,41], and by the expression and activity of FPGS in reticulocytes [42,43,44]. Clinical and lifestyle factors like BMI, smoking, and alcohol consumption cannot fully explain the variations seen in patients. Models including clinical, lifestyle, and SNPs of different enzymes in the folate pathway could also not explain the variations in full [9,10,11]. Models including erythrocyte folate correcting for MTX dose, age, and a SNP for FPGS performed similarly, with R^2^ between 0.1 and 0.15 for MTX-PG_1–3_ and 0.16 for MTX-PG_2–5_ [11]. Additionally, one of the major factors that determines MTX-PG concentrations is FPGS enzyme activity, which was not included in this model. MTX-PG accumulation occurs in the precursor cells of erythropoiesis, as mature erythrocytes lack FPGS activity [45]. Erythroid precursors, however, are challenging to isolate due to their scarcity in peripheral circulation, the necessity of invasive bone marrow sampling, and their rapid maturation to erythrocytes. Erythrocyte folate could be used as a surrogate marker for FPGS activity, as studies suggest erythrocyte folate concentration influences MTX response and MTX-PG concentrations [18,46]. A recent study additionally suggested that lower plasma folate level was associated with risk of MTX discontinuation [47]. Uptake of MTX into the cells is another factor which has not been studied yet. Saturation of RFCs can occur in high-dose MTX treatment, while in IMIDs the dose is generally below the maximum capacity of RFC (K_m_ of 5 µmol/L) [7]. RFC expression and activity in erythroid precursor cells could play a role for the pharmacokinetics/pharmacodynamics of MTX-PG accumulation.

## 4. Materials and Methods

### 4.1. Patient Population

We included 567 patients diagnosed with RA (*n* = 287), JIA (*n* = 115), CD (*n* = 57), sarcoidosis (*n* = 71), or ped-ALL (*n* = 37), with MTX-PG_2–5_ quantified after 3 months of MTX treatment. RA patients were from the Methotrexate-Rotterdam (MTX-R), treatment in Rotterdam Early Arthritis Cohort (tREACH; trial ID: ISRCTN26791028) and Methotrexate Monitoring clinical study (MeMo; trial ID: NTR7149) studies. Dose escalation decisions in MTX-R and MeMo were made by the attending physician. tREACH patients started treatment at 15 mg/week escalating to 25 mg/week over 3 weeks, as per the study protocol. Oral and subcutaneous (S.C.) MTX use were permitted in the RA cohorts [7,48]. JIA (trial ID: ISRCTN13524271) patients started with oral or S.C. MTX [49]. Sarcoidosis patients included in the ILD BIOBANK at St. Antonius Hospital, treated with oral MTX between 2018 and 2021 were included retrospectively. Patients started MTX at 10 mg/week for 2 weeks, escalating to 12.5 mg/week after 2 weeks and finally to 15 mg/week until response assessment. CD patients (CCMO trial register: NL67718.029.18) started on S.C. MTX and could taper to 15 mg/week after 8 weeks [5,47]. In IMIDs, time between MTX administration and sample drawn was not protocolized; therefore, samples were collected at regular patient visits. MTX-PG concentrations were quantified at variable times within 1 week of the previous MTX dose. Ped-ALL patients in the intensification phase were treated with high-dose MTX, according to the DCOG ALL-11 protocol (CCMO trial register: NL50250.078.14) [14]. All patients received 4 doses of 5000 mg/m^2^ I.V. MTX in 24 h every 2 weeks, with oral 6-mercaptopurine, and intrathecal (I.Th.) MTX was administered prior to every high-dose cycle. MTX-PG concentrations were quantified 2 weeks after the 4th dose of I.V. MTX (corresponding to approximately 2.5 months after start of high-dose MTX therapy). Predniso(lo)ne use was permitted for JIA, RA, and sarcoidosis with doses protocolized in the respective studies. RA patients were permitted to use disease-modifying anti-rheumatic drugs (DMARDs; hydroxychloroquine (HCQ) with(out) sulfasalazine (SSZ)) as per the respective study protocols. Ped-ALL patients on asparaginase therapy were included (*n* = 8). IMID patients received folic acid supplementation once per week, dosed as per individual study protocols, while ped-ALL patients received folinic acid rescue (15 mg/m^2^/dose) at 42, 48, and 54 h after the start of high-dose MTX administration. Exclusion criteria included unknown starting MTX dose, concomitant or unknown status of baseline use of biologics in IMID patients (*n* = 9; RA and *n* = 3; JIA), and no measurable concentrations of MTX-PG (*n* = 1, RA). All ethical approvals were obtained in the original studies with their respective local ethical boards.

### 4.2. Study Variables

MTX-PG_2–5_ were quantified by a validated UPLC-MS/MS method as previously described and the concentration were expressed in nmol/L packed erythrocytes abbreviated as nmol/L [50]. We focused our analysis on the active metabolites MTX-PG_2–5_, since MTX-PG_1_ is the native form of MTX, with variable concentrations with high inter-patient variability. Possible determinants were selected based on the literature and included age (in years), sex, baseline use of disease-modifying anti-rheumatic drugs (DMARDs) (HCQ, SSZ; dichotomous-yes/no), baseline use of predniso(lo)ne (dichotomous, yes/no), 3-month use of folic acid (folic/folinic acid; folinic acid dose was normalized to mg/week) dosed >5 mg/week (dichotomous, yes/no), smoking assessed via questionnaires (dichotomous, current/former vs. never), body mass index (BMI, in kg/m^2^), route of MTX administration (categorical—oral, S.C., and I.V./I.Th.), and starting dose of MTX (in mg/week) [9,10,11,18]. MTX dose in JIA and ped-ALL were body surface area-based and was converted to mg/week using the Mosteller formula for BSA dosing. Additionally, IMID patients were categorized as low-dose, while ped-ALL were categorized as high-dose. Estimated GFR (eGFR) at 3 months (in mL/min/1.73 m^2^) was recalculated according to the CKD-EPI (2021 update) and using the bedside Schwartz Formula for JIA and ped-ALL [51]. Ped-ALL and JIA cohorts were assumed to be non-smokers due to their young age.

### 4.3. Statistical Analysis

All analysis were performed in R (version 4.3.2; R Foundation for statistical computing, Vienna, Austria). Between-group differences were assessed with pairwise Wilcox rank-sum test and *p*-values adjusted using Bonferroni procedure. Missing data per variable was < 30% and was handled using multiple imputation by chained equations (MICE; m = 15) using “mice” package (version 3.16.0) in R. Multiple imputation was chosen for its robustness in handling outliers, while preserving the data distribution [52]. We constructed multivariate linear regression models for individual MTX-PG_2–5,_ as well as with the sum of long-chain MTX-PGs (MTX-PG_3–5sum_) for the full patient cohort, as well as a subset of IMIDs patients. MTX-PG_4_, MTX-PG_5_, and MTX-PG_3–5sum_ were not normally distributed and thus log-transformed prior to modelling; reported effect estimates were back-transformed for interpretation. The models were checked for multicollinearity among predictors using variance inflation factors (VIF). Factors with a VIF above 5 were removed from the models. Model diagnostics were performed on the imputed datasets using stripplots to compare the imputed and observed values, as well as examination of residuals (of the first imputed dataset) for both log-transformed and untransformed outcomes. The β-estimates of the models were back-transformed for interpretations where needed. All plots were constructed in R using “ggplot2” package.

## 5. Conclusions

Concentration of MTX-PGs in erythrocytes is influenced by route of administration, concomitant prednisone, DMARD and folic/folinic acid use, age, and BMI, largely independent of underlying disease. High-dose I.V./I.Th. MTX in ped-ALL patients led to markedly higher MTX-PG_4–5_ concentrations. Low-dose S.C. MTX administered in IMIDs showed comparable MTX-PG_2–5sum_ concentration to high-dose I.V./I.Th. MTX administered in ped-ALL patients. Parenteral MTX administration during the first three months of treatment was associated with higher MTX-PG concentrations, while older age or higher BMI was associated with lower concentrations, identifying patient subgroups in whom MTX-PG exposure might differ. These findings highlight populations that may warrant further prospective investigations on MTX-PG-guided dosing to improve clinical outcomes. Integrating these determinants into clinical practice provides a rationale for personalized MTX therapy, optimizing dosing strategies, and improving treatment outcomes across diverse inflammatory and hematological conditions.

## Figures and Tables

**Table 1:** Baseline patient characteristics. RA: Rheumatoid arthritis, JIA: Juvenile idiopathic arthritis, CD: Crohn’s disease, ped-ALL: pediatric acute lymphoblastic leukemia, I.Th.: Intrathecal, I.V.: Intravenous, DMARD: Disease-modifying anti-rheumatic drug.

	RA	JIA	Sarcoidosis	CD	Ped-ALL
Number of patients (%)	287 (50.6)	115 (20.3)	71 (12.5)	57 (10.1)	37 (6.5)
Age (year); mean ± SD	53.34 ± 14.59	11 ± 4.29	51.8 ± 9.60	54.5 ± 13.2	6.40 ± 4.25
Female; *n* (%)	209 (70.6%)	71 (60.2%)	29 (40.8%)	37 (64.9%)	14 (37.8%)
Baseline GFR (mL/min/1.73 m^2^), mean ± SD	83.25 ± 19.75	*N.A.*	91.99 ± 19.12	96.22 ± 14.48	*N.A.*
Body Surface Area (BSA) (m^2^); median (IQR)	1.86 (1.8–2.03)	1.34 (0.94–1.62)	2.04 (1.9–2.24)	1.9 (1.73–2.04)	0.74 (0.65–1.05)
**MTX**					
Starting dose (mg/week)	20.05 ± 5.92	12.52 ± 3.89	10 ± 0	24.82 ± 1.32	976.47 ± 394.2
Cumulative dose till 3 months (mg) ^$^; mean ± SD	86.77 ± 101.68	112.9 ± 34.61	120.14 ± 1.93	178.33 ± 7.65	58,588.11 ± 23,652
I.Th. dose (mg/dose)	*N.A.*	*N.A.*	*N.A.*	*N.A.*	11.35 ± 1.16
Folic acid dose (mg/week)—baseline ^$$^	8.71 ± 2.19	5	10	5	*N.A.*
Folinic acid dose after MTX infusion (mg/week) ^$$$^	*N.A.*	*N.A.*	*N.A.*	*N.A.*	13.45 ± 5.43
**Route of administration baseline; *n* (%)**					
Oral	262 (91.3%)	113 (98.3%)	71 (100%)	*N.A.*	*N.A.*
Subcutaneous	25 (8.5%)	2 (1.7%)	*N.A.*	57 (100%)	*N.A.*
I.V. with I.Th.	*N.A.*	*N.A.*	*N.A.*	*N.A.*	37 (100%)
**Baseline use of comedication**					
Starting dose—Predniso(lo)ne	12.92 ± 5.92		10		NA
Baseline use of biologics (**No**); *n* (%)	287 (100%)	115 (100%)	71 (100%)	57 (100%)	29 (78%)
Baseline use of DMARDs (**No**); *n* (%) ^#^	135 (47%)	114 (96.6%)	71 (100%)	56 (98.2%)	*N.A.*

^$^ 2.5 months for ped-ALL, corresponding to the 4th MTX infusion dose. ^$$^ For sarcoidosis, folic acid was dosed at 5 mg twice a week. ^$$$^ ped-ALL patients received folinic acid rescue (15 mg/m^2^/dose) at 42, 48, and 54 h after the start of HDMTX administration. ^#^ DMARDs included hydroxychloroquine and sulfasalazine; *N.A.*—not applicable.

**Table 2:** The effect measures and *p*-values from the multivariate linear regression analysis across all cohorts. The effect measures for MTX-PG_2,3_ is β, while for MTX-PG_4,5_ and MTX-PG_3–5sum_ the effect measure is e^β^. Significant values are highlighted in bold. MTX-PG: Methotrexate polyglutamates, BMI: Body mass index, S.C.: Subcutaneous, I.V./I.Th.: Intravenous/intrathecal, DMARD: Disease-modifying anti-rheumatic drug, GFR: Glomerular filtration rate. * *p*-value < 0.05, ** *p*-value ≤ 0.01, *** *p*-value ≤ 0.001.

Factors	MTX-PG_2_	MTX-PG_3_	MTX-PG_4_	MTX-PG_5_	MTX-PG_3–5sum_
Age (years)	0.16 (0.01) **	0.36 (*p* < 0.001) ***	**1.01** (0.002) **	**1.01** (0.003) **	**1.01** (0.002) **
BMI (kg/m^2^)	−0.33 (0.01) **	−0.61 (0.002) **	0.98 (0.06)	1.00 (0.71)	0.98 (0.001) **
Sex (male)	0.67 (0.58)	−0.03 (0.99)	1.00 (0.96)	0.97 (0.77)	1.02 (0.74)
Smoking (Current/former)	0.80 (0.62)	1.05 (0.67)	1.11 (0.33)	1.05 (0.70)	1.06 (0.51)
Starting MTX dose (mg/week)	4.63 × 10^−4^ (0.85)	−0.005 (0.23)	1.00 (0.22)	1.00 (0.124)	1.00 (0.21)
Baseline route (S.C.)	−5.77 (0.01) **	11.84 (0.001) ***	2.25 (*p* < 0.001) ***	2.19 (*p* < 0.001) ***	1.75 (*p* < 0.001) ***
Baseline route (I.V./I.Th.)	−1.66 (0.80)	22.26 (0.04) *	18.65 (*p* < 0.001) ***	86.48 (*p* < 0.001) ***	6.75 (*p* < 0.001) ***
Baseline use of prednisone (yes)	2.81 (0.41)	8.59 (0.07)	**1.83** (0.007) **	3.65 (*p* < 0.001) ***	**1.59** (0.004) **
Baseline use of DMARD (yes)	−4.62 (0.001) ***	−5.25 (0.020) *	0.88 (0.18)	**0.78** (0.02) *	0.87 (0.07)
Use of Folic/folinic acid (>5 mg/week)	2.04 (0.27)	**7.07** (0.008) *	**1.43** (0.002) **	**1.31** (0.02) *	1.40 (*p* < 0.001) ***
Estimated GFR (mL/min/1.73 m^2^)	−0.02 (0.74)	−0.10 (0.14)	1.00 (0.14)	1.00 (0.64)	1.00 (0.26)
R^2^	0.09	0.26	0.40	0.58	0.32

## Data Availability

The data presented in this study are available on request from the corresponding author. The data are not publicly available due to privacy and ethical restrictions (patient confidentiality).

## Supplementary Materials

The following supporting information can be downloaded at: https://www.mdpi.com/article/10.3390/ph19020267/s1, Table S1: MTX-PG concentrations stratified by disease, Table S2: Variables included in modelling and imputation, Table S3: Effect measures (β, eβ) and *p*-value across MTX-PG models (in subset of IMID patients), Table S4: Effect measures (β, eβ) and *p*-value across MTX-PG models (with cumulative dose); Figure S1: Proportion of MTX-PGs normalized to MTX-PG2–5sum, Figure S2: Missingness Summary plot, Figure S3: Representative stripplots, Figure S4: Representative residual plots.

## Abbreviations

The following abbreviations are used in this manuscript:

MTX	Methotrexate
MTX-PG	Methotrexate polyglutamate
IMID	Immune-mediated inflammatory diseases
Ped-ALL	Pediatric acute lymphoblastic leukemia
RA	Rheumatoid arthritis
JIA	Juvenile idiopathic arthritis
CD	Crohn’s diseases
FPGS	Folyl-polyglutamate synthetase
TDM	Therapeutic drug monitoring
SNP	Single nucleotide polymorphism
S.C.	Subcutaneous
I.V.	Intravenous
I.Th.	Intrathecal
DMARD	Disease-modifying anti-rheumatic drugs
HCQ	Hydroxychloroquine
SSZ	Sulfasalazine
UPLC-MS/MS	Ultra-Performance Liquid Chromatography-Tandem Mass Spectrometry
BMI	Body mass index
BSA	Body surface area
eGFR	Estimated glomerular filtration rate
CKD-EPI	Chronic Kidney Disease Epidemiology Collaboration
MICE	Multiple imputation by chained equations
SD	Standard deviation
IQR	Interquartile range
AO	Aldehyde oxidase
RFC	Reduced folate carrier
Km	Michaelis constant
