# Supplementary material for: Drivers of Methotrexate Polyglutamate Concentration in Erythrocytes: Insights from Immune-Mediated Inflammatory Diseases and Pediatric Acute Lymphoblastic Leukemia
Source: Pharmaceuticals (Basel). 2026 Feb 4;19(2):267. doi: 10.3390/ph19020267 (PMC12943293; doi:10.3390/ph19020267)
Supplement: Supplementary file 1 [file pharmaceuticals-19-00267-s001.zip › pharmaceuticals-4067562-supplementary.pdf]

Table S1. MTX-PG concentrations stratified by disease: Median (IQR) concentrations of individual MTX-PG_{2-5} and MTX-PG_{2-5sum} and MTX-PG_{3-5sum} stratified by disease. MTX-PG: Methotrexate polyglutamate, RA: rheumatoid arthritis, JIA: Juvenile idiopathic arthritis, CD: Crohn's disease, Ped-ALL: Pediatric acute lymphoblastic leukemia.

	**MTX-PG_2**	**MTX-PG_3**	**MTX-PG_4**	**MTX-PG_5**	**MTX-PG_{2-5sum}**	**MTX-PG_{3-5sum}**
**RA**	21.62 (15.51–28.14)	46.95 (31.23–59.37)	16.06 (8.69–29.28)	4.98 (2.63–11.2)	91.18 (63.8–122.8)	68.4 (41.86–95.84)
**JIA**	18.7 (14.9–24.15)	23 (13.85–33.5)	4.4 (2.25–8.65)	0.7 (0.3–1.75)	48.3 (34.1–68.5)	27.7 (16.1–43.8)
**Sarcoidosis**	32.3 (20.99–44.42)	51.04 (32.9–69.48)	15.41 (10.37–24.84)	6.96 (4.13–9.65)	107.17 (77.52–149.38)	71.92 (49.48–100.37)
**CD**	20.66 (15.73–25.98)	54.27 (40.46–73.87)	29.8 (20.02–51.46)	12.04 (7.25–20.52)	113.27 (92.01–179.06)	94.4 (72.51–148)
**Ped-ALL**	10.8 (6.5–22.7)	33 (27.2–41.1)	48.7 (34.8–71.1)	42.9 (24.4–53.6)	141.3 (101.6–181.7)	124.1 (87.4–160.5)
**Overall**	21 (15.4–28.56)	41.3 (26.32–57.4)	15.42 (6.81–29.97)	5.3 (1.9–12.04)	88.71 (55.16–124.86)	63.3 (34.76–97.49)

Table S2. Variables included in modeling and imputation: Variables were included as either "predictor; auxiliary" variables (included in model construction and to impute the missing data) auxiliary variables (not included in model constructions, used only for imputation of missing data) or as predictors (included in the model and were imputed). "-" represent data that did not have any missing. BMI: Body mass index, MTX: Methotrexate, DMARD: disease modifying anti-rheumatic drugs; GFR: Glomerular filtration rate; ESR: erythrocyte sedimentation rate, CRP: c-reactive protein.

**Variable**	**Role in analysis**	**Missingness (%)**	**imputed**
Age	Predictor; Auxiliary	-	No
BMI	Predictor	23	Yes
Sex	Predictor; Auxiliary	-	No
Smoking	Predictor	19	Yes
Route of administration	Predictor; Auxiliary	-	No
Starting MTX dose	Predictor; Auxiliary	-	No
Baseline use of prednisone	Predictor	22	Yes
Baseline use of DMARD	Predictor; Auxiliary	-	No
Use of folic/folinic acid >5mg/week	Predictor	1	Yes
Estimated GFR	Predictor	8	No
ESR	Auxiliary	28	No
Disease duration	Auxiliary	28	No
CRP	Auxiliary	17	No
Height	Auxiliary	21	No

Table S3. Effect measures and p-value across MTX-PG models (in subset of IMID patients): The effect measures for MTX-$PG_{2,3}$ is β, while for MTX-$PG_{4,5}$ and MTX-$PG_{3\text{-}5sum}$, the effect measure is e^{β}. MTX-PG: Methotrexate polyglutamates, BMI: body mass index, S.C.: subcutaneous, DMARD: disease modifying anti-rheumatic drug, GFR: glomerular filtration rate. * p-value <0.05, ** p-value ≤0.01, *** p-value ≤0.001.

Factors	MTX-PG_2	MTX-PG_3	MTX-PG_4	MTX-PG_5	MTX-$PG_{3\text{-}5\text{-}sum}$
Age (Years)	**0.18 (0.001)** **	**0.39 (p<0.001)** ***	**1.01 (0.001)****	**1.02 (0.002)****	**1.01 (p<0.001)** ***
BMI (kg/m²)	**-0.36 (0.003) ****	**-0.59 (0.005)****	0.98 (0.068)	1.00 (0.95)	**0.98 (0.002)*** *
Sex (male)	0.81 (0.49)	-0.33 (0.87)	0.99 (0.98)	0.98 (0.82)	1.01 (0.85)
Smoking (Current/former)	1.41 (0.35)	0.98 (0.72)	1.09 (0.421)	1.07 (0.61)	1.06 (0.59)
Baseline route (S.C.)	-1.89 (0.39)	**14.99 (p<0.001)**** *	**2.09 (p<0.001)** ***	**2.30 (p<0.001)** ***	**1.84 (p<0.001)** ***
Starting MTX dose (mg/week)	**-0.53 (p<0.001)** ***	-0.29 (0.10)	1.01 (0.1)	1.00 (0.65)	1.00 (0.77)
Baseline use of prednisone (Yes)	1.91 (0.50)	6.14 (0.29)	**1.69 (0.010)***	**2.93 (p<0.001)** ***	**1.42 (0.04)***
Baseline use of DMARD (Yes)	-1.50 (0.29)	-3.40 (0.18)	0.81 (0.06)	**0.78 (0.02)***	0.86 (0.09)
Use of Folic acid >5mg/week (Yes)	**4.24 (0.01)***	**8.90 (0.005)****	**1.38 (0.01)****	1.37 (0.08)	**1.46 (0.001)****
Estimated GFR	0.01 (0.90)	-0.08 (0.24)	0.99 (0.160)	1.00 (0.58)	1.00 (0.28)
R2	0.15	0.26	0.35	0.49	0.29

Table S4. Effect measures and p-value across MTX-PG models (with cumulative dose): The effect measures (p-values) from the multivariate linear regression analysis incorporating cumulative MTX dose across all patients. The effect measures for $MTX\text{-}PG_{2,3}$ is β, while for $MTX\text{-}PG_{4,5}$ and $MTX\text{-}PG_{3\text{-}5sum}$, the effect measure is e^{β}. Starting MTX dose was replaced by cumulative MTX dose in this model. MTX-PG: Methotrexate polyglutamates, BMI: body mass index, S.C.: subcutaneous, I.V./I.Th.: intravenous/intrathecal, DMARD: disease modifying anti-rheumatic drug, GFR: glomerular filtration rate. * p-value <0.05, ** p-value ≤0.01, *** p-value ≤0.001.

**Factors**	**$MTX\text{-}PG_2$**	**$MTX\text{-}PG_3$**	**$MTX\text{-}PG_4$**	**$MTX\text{-}PG_5$**	**$MTX\text{-}PG_{3\text{-}5\text{-}sum}$**
Age (years)	**0.17 (0.008)****	**0.36 (0.003)*****	**1.01 (0.002)***	**1.01 (0.003)***	**1.01 (0.003)****
BMI (kg/m²)	**-0.34 (0.008)****	**-0.58 (0.01)****	0.98 (0.07)	1.00 (0.81)	**0.98 (0.005)****
Sex (male)	0.77 (0.53)	0.05 (0.98)	1.00 (0.96)	0.97 (0.77)	1.03 (0.71)
Smoking (Current/former)	1.10 (0.47)	0.73 (0.77)	1.11 (0.33)	1.07 (0.60)	1.06 (0.56)
Cumulative MTX dose (mg)	1.62×10^{-5} (0.72)	-8.55×10^{-5} (0.25)	1.00 (0.18)	1.00 (0.11)	1.00 (0.19)
Baseline route (S.C.)	**-5.97 (0.007)****	**12.00 (p<0.001)*****	**2.23 (p<0.001)*****	**2.18 (p<0.001)*****	**1.75 (p<0.001)*****
Baseline route (I.V./I.Th.)	-3.14 (0.64)	**21.95 (0.04)***	**19.65 (p<0.001)*****	**89.51 (p<0.001)*****	**6.93 (p<0.001)*****
Baseline use of prednisone (yes)	2.56 (0.43)	8.39 (0.11)	**1.81 (0.005)***	**3.44 (p<0.001)*****	**1.55 (0.01)****
Baseline use of DMARD (yes)	**-4.67 (0.001)****	**-5.24 (0.02)***	0.88 (0.18)	**0.78 (0.02)***	0.87 (0.08)
Use of Folic/folinic acid (>5mg/week)	2.08 (0.24)	**7.06 (0.007)****	**1.43 (0.002)***	1.32 (0.05)	**1.39 (p<0.001)*****
Estimated GFR (mL/min/1.73m²)	-0.004 (0.93)	-0.10 (0.15)	1.00 (0.14)	1.00 (0.60)	1.00 (0.32)
R^2	0.09	0.26	0.40	0.58	0.32

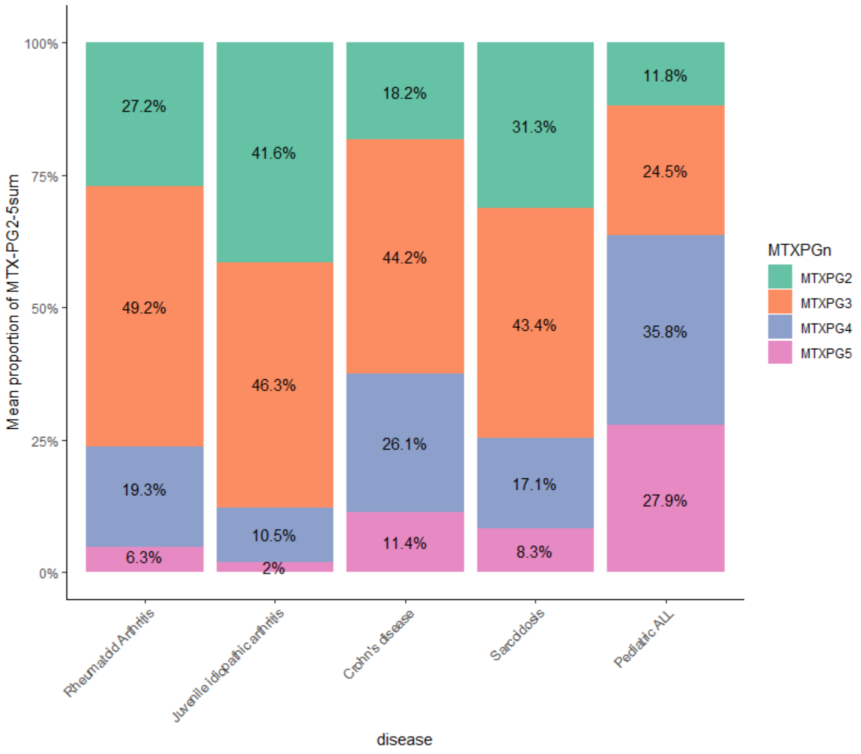

Figure S1. Proportion of MTX-PGs normalized to MTX-PG2-5sum. Individual MTX-PGs were normalized to MTX-PG2-5sum, stratified by disease and their percentags were plotted. MTX-PG: Methotrexate polyglutamates.

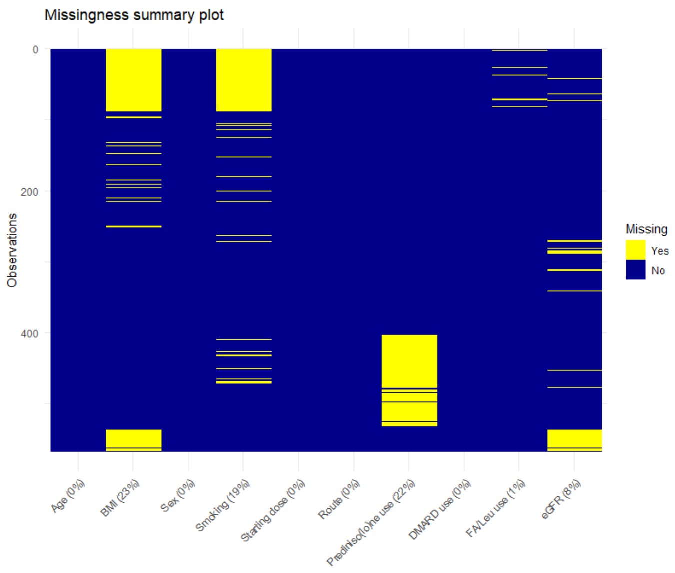

Figure S2. Missingness Summary plot: The missingness summary plot depicts the percentage missing data among the predictors. BMI: body mass index, DMARD: disease modifying anti-rheumatic drug, FA/Leu: folic/folinic acid, eGFR: estimated glomerular filtration rate.

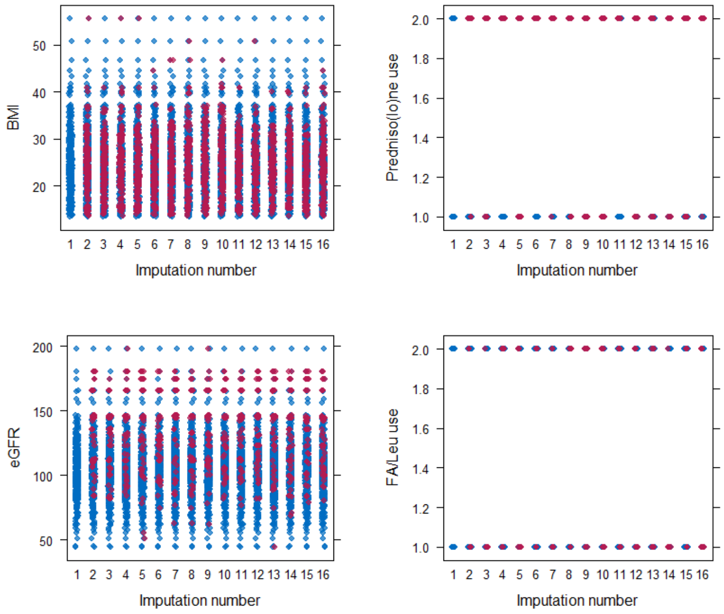

Figure S3. Representative stripplots. Stripplots were constructed for the missing variables for MTX-PG2 is shown here. The stripplots show good agreement between the observed and imputed values. BMI: body mass index, eGFR: estimated glomerular filtration rate, FA/Leu: folic/folinic acid.

A

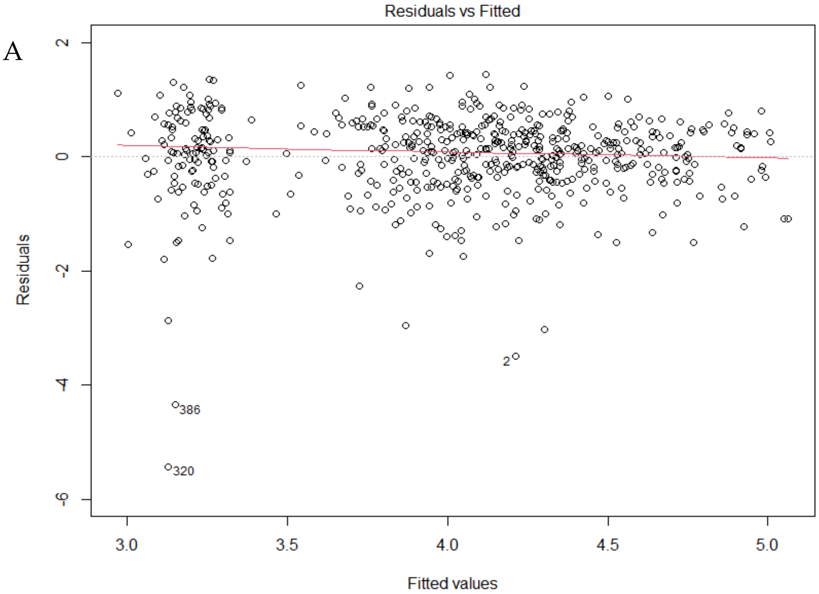

B

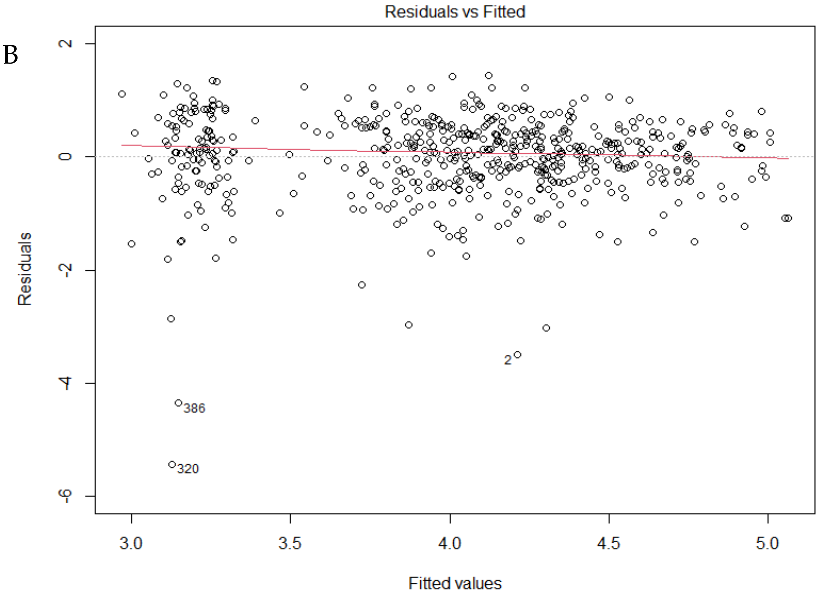

Figure S4. Representative residual plots. Representative residual plots from the first imputed dataset (m=1) is provided below. As some of our outcomes were log-transofrmed, the residual plots are shown for A: Not log-transfromed outcome (MTX-PG3), B: Log-transformed outcome (MTX-PG$_{3\text{-}5Sum}$). MTX-PG: Methotrexate polyglutamates.
